# Pain Mechanisms in Fibromyalgia: An Integrative Narrative Review of Central, Peripheral, Neuroimmune, and Psychobiological Factors

**DOI:** 10.3390/biomedicines14092012

**Published:** 2026-09-08

**Authors:** Filipa Martins-Alves, Armando Almeida

**Affiliations:** 1 Department of Psychiatry, Alto Ave Local Health Unit, 4835-044 Guimarães, Portugal; filipaalves@ulsaave.min-saude.pt; 2 Life and Health Sciences Research Institute (ICVS), School of Medicine, University of Minho, 4710-057 Braga, Portugal; 3 ICVS/3B’s—PT Government Associate Laboratory, 4710-057 Braga, Portugal; 4 ICVS/3B’s—PT Government Associate Laboratory, 4805-017 Guimarães, Portugal

**Keywords:** fibromyalgia, nociplastic pain, central sensitization, descending pain modulation, small-fiber pathology, neuroinflammation

## Abstract

**Background/Objectives:** Fibromyalgia is a chronic pain condition characterized by widespread musculoskeletal pain, fatigue, sleep disturbance, cognitive dysfunction, and multisensory hypersensitivity. It is increasingly conceptualized as a heterogeneous nociplastic pain condition in which altered nociceptive processing interacts with dysfunctional pain regulation, neuroimmune mechanisms, and variable peripheral contributions. This integrative narrative review aims to synthesize current evidence on the major mechanisms underlying pain in fibromyalgia, with particular emphasis on central sensitization, descending pain modulation, neurochemical dysregulation, small-fiber pathology, neuroimmune processes, and psychobiological modulators. **Methods:** An integrative narrative review was conducted using iterative, mechanism-oriented searches of the biomedical literature, primarily in PubMed/MEDLINE and complemented by targeted bibliographic searches and reference tracking. Research published up to July 2026 was considered, with emphasis on human mechanistic studies, systematic reviews, meta-analyses, and landmark experimental evidence relevant to the major pathophysiological domains of fibromyalgia. **Results:** Central sensitization and altered nociceptive gain remain prominent mechanisms of pain amplification in fibromyalgia, but they do not fully account for the clinical phenotype. Evidence also supports impaired and heterogeneous descending pain modulation, neurochemical imbalance, neuroimmune activation, autonomic and stress-system dysregulation, and peripheral contributions, including small-fiber pathology in a substantial subgroup of patients. These mechanisms appear to interact rather than operate independently, while cognitive and emotional factors further modulate symptom severity, persistence, and functional impact. **Conclusions:** Fibromyalgia is best understood as a heterogeneous nociplastic pain syndrome arising from partially overlapping central, peripheral, neuroimmune, autonomic, and psychobiological mechanisms whose relative contribution varies across patients. Recognizing this mechanistic heterogeneity may improve phenotypic stratification, biomarker development, and the design of more individualized, mechanism-informed therapeutic strategies.

## 1. Introduction

Fibromyalgia is a chronic pain condition characterized by widespread musculoskeletal pain accompanied by fatigue, sleep disturbance, cognitive dysfunction, and multisensory hypersensitivity [1,2,3,4]. Its clinical expression is markedly heterogeneous and extends well beyond pain alone, with substantial effects on daily functioning, psychosocial well-being, and health care utilization [1,5]. This multidimensional phenotype has contributed to a progressive shift in the way fibromyalgia is conceptualized, from a disorder defined primarily by widespread tenderness toward one increasingly understood in terms of altered nociceptive processing and pain regulation [1,5,6].

Historically, the 1990 American College of Rheumatology (ACR) classification criteria defined fibromyalgia by chronic widespread pain together with pain on palpation at least 11 of 18 designated tender points [3,4,5,7]. These criteria standardized case identification and facilitated clinical research, but they also placed considerable emphasis on a physical examination construct that captured only part of the clinical syndrome. Subsequent work demonstrated that pain hypersensitivity in fibromyalgia is not restricted to predefined tender-point locations and that fatigue, nonrestorative sleep, cognitive symptoms, and broader functional impairment represent integral components of the disorder [1,2,3,4,5,6]. Revised ACR criteria consequently reduced the importance of tender-point examination and gave greater weight to widespread pain distribution and symptom severity [3,4,5,8,9]. In parallel, mechanistic research increasingly shifted attention away from local musculoskeletal pathology toward altered processing and regulation of nociceptive information within the nervous system [1,6,10,11].

Central sensitization became a major framework for interpreting this transition. It refers to increased responsiveness of nociceptive neurons within the central nervous system, resulting in enhanced nociceptive gain and clinical manifestations such as hyperalgesia and allodynia [12]. In fibromyalgia, widespread pain hypersensitivity, enhanced temporal summation, prolonged painful after-sensations, and neuroimaging evidence of altered pain processing have provided substantial support for central amplification as an important pathophysiological mechanism [1,6,10,11]. However, central sensitization should not be regarded as a complete explanation of fibromyalgia, nor should it be used interchangeably with the more recent concept of nociplastic pain.

Nociplastic pain was introduced as a third mechanistic pain descriptor, alongside nociceptive and neuropathic pain. It refers to pain arising from altered nociception despite no clear evidence of actual or threatened tissue damage causing activation of peripheral nociceptors and no evidence of a disease or lesion of the somatosensory system that would explain the pain [3,11,13,14]. Fibromyalgia is widely regarded as a prototypical clinical condition within this framework [3,5,13,14]. Importantly, nociplastic pain and central sensitization describe different levels of analysis: nociplastic pain is a clinical descriptor, whereas central sensitization is a neurophysiological mechanism [3,11,12,14]. Central sensitization may contribute substantially to the nociplastic phenotype of fibromyalgia, but nociplastic pain does not imply that a single central mechanism accounts for all symptoms or all patients [12,13,14].

This distinction is particularly relevant because contemporary evidence supports fibromyalgia as a biologically heterogeneous condition [5,15,16]. Patients do not show identical sensory profiles, the same degree of central pain amplification, or the same efficiency of descending pain modulation [5,15,16,17,18,19]. Peripheral abnormalities, including small-fiber pathology and persistent nociceptive input, have been identified in subsets of patients, while neuroimmune signaling, autonomic and stress-system dysregulation, sleep disturbance, and cognitive-emotional processes may further shape pain severity and persistence [1,4,6,16,17,18,19,20,21]. These mechanisms may coexist and interact, with their relative contribution varying across individuals and potentially over time. Evidence of central amplification does not exclude relevant peripheral input, just as the presence of small-fiber abnormalities in some patients does not require fibromyalgia as a whole to be reclassified as a peripheral neuropathy [6,13,14,17,18,19]. Current diagnostic frameworks also allow fibromyalgia to occur as a primary nociplastic condition or in association with other rheumatological or chronic pain disorders; the presence of a coexisting disease neither excludes fibromyalgia nor, by itself, establishes that peripheral nociceptive input from that disease accounts for the full clinical phenotype [3,8,9,13].

This heterogeneity has important implications for both mechanistic interpretation and future patient stratification. Rather than assuming that all individuals share a single pathogenic pathway, current evidence favors a model in which central amplification provides a major organizing mechanism while descending modulatory dysfunction, neurochemical alterations, peripheral nociceptive input, small-fiber pathology, neuroimmune processes, autonomic and stress-system disturbances, and cognitive-emotional modulation contribute with different relative weights [1,4,6,16,17,18,19,20]. At present, these patterns should be regarded as partially overlapping mechanistic configurations rather than as prospectively validated biological subtypes [16,17,18,19].

Accordingly, this integrative narrative review examines the major mechanisms proposed to underlie pain in fibromyalgia, with particular emphasis on central sensitization, descending pain modulation, neurochemical dysregulation, peripheral nociceptive contributions and small-fiber pathology, neuroimmune mechanisms, stress and autonomic regulation, and psychological and cognitive modulators. By considering both convergent evidence and areas of uncertainty, we aim to develop an integrated framework that accounts for the biological heterogeneity of fibromyalgia and clarifies how interacting mechanisms may contribute to different clinical phenotypes and, ultimately, to mechanism-informed patient stratification.

## 2. Methods

This integrative narrative review was developed through iterative, mechanism-oriented searches of the biomedical literature. The overall approach was informed by established methodological principles for narrative and integrative reviews, particularly the need for transparent literature searching, critical selection of evidence, integration of heterogeneous study designs, and explicit distinction between evidence synthesis and formal systematic review methodology [22,23]. Searches were conducted primarily in PubMed/MEDLINE and were complemented by targeted searches of bibliographic databases, journal and publisher websites, and reference lists of relevant articles. The literature search was progressively updated during manuscript preparation, with the final update performed in July 2026.

Searches were organized around the major mechanistic domains addressed in the review. The term *fibromyalgia* was combined with relevant keywords and related terms, including *nociplastic pain*, *central sensitization*, *temporal summation*, *wind-up*, *descending pain modulation*, *conditioned pain modulation*, *diffuse noxious inhibitory controls*, *pain facilitation*, *periaqueductal gray*, *rostral ventromedial medulla*, *locus coeruleus*, *dorsal reticular nucleus*, *glutamate*, *substance P*, *serotonin*, *noradrenaline*, *dopamine*, *endogenous opioids*, *peripheral sensitization*, *muscle nociception*, *small-fiber pathology*, *small-fiber neuropathy*, *intraepidermal nerve fiber density*, *corneal confocal microscopy*, *dorsal root ganglia*, *satellite glial cells*, *neuroinflammation*, *microglia*, *astrocytes*, *cytokines*, *autoantibodies*, *autonomic dysfunction*, *heart rate variability*, *hypothalamic–pituitary–adrenal axis*, *stress*, *sleep*, *sex hormones*, *catastrophizing*, *hypervigilance*, *cognitive dysfunction*, and *psychological modulation*. Searches were refined iteratively as additional mechanistic themes and relevant studies emerged.

No lower publication-date limit was imposed, because seminal studies were considered necessary for tracing the development of several mechanistic concepts, including central sensitization, diffuse noxious inhibitory controls, descending pain modulation, neurochemical abnormalities, and small-fiber pathology. However, particular emphasis was placed on recent publications, especially studies and reviews published during the last decade and, where available, evidence published between 2021 and 2026.

Priority was given to peer-reviewed human studies directly addressing fibromyalgia, including psychophysical, quantitative sensory testing, neuroimaging, neurochemical, histological, autonomic, immunological, and clinical investigations. Systematic reviews and meta-analyses were used to identify areas of convergence, heterogeneity, and uncertainty, whereas landmark experimental studies were retained when they provided mechanistic foundations not adequately captured by more recent work. Relevant animal and preclinical studies were included selectively when required to explain established mechanisms or when direct human evidence was limited, but mechanistic conclusions were distinguished from findings demonstrated specifically in patients with fibromyalgia.

Reference lists of key reviews and primary studies were examined to identify additional relevant publications. When a mechanistic claim was supported by both review-level and primary evidence, preference was given to the original study for specific experimental findings, while recent reviews were used to provide broader context and to assess consistency across the literature. Particular attention was paid to distinguishing association, biological plausibility, and demonstrated causality, especially for neurochemical abnormalities, neuroimmune mechanisms, small-fiber pathology, autonomic dysfunction, and psychological factors.

Studies were selected on the basis of their relevance to the mechanistic questions addressed in the review, methodological quality, contribution to current understanding, and ability to illustrate areas of agreement or controversy. Consistent with the purpose of an integrative narrative review, the objective was to synthesize heterogeneous mechanistic evidence rather than to estimate pooled effects or answer a narrowly predefined clinical question [22,23]. No formal systematic-review protocol, prospective registration, PRISMA-based study-selection process, or meta-analysis was therefore undertaken. Accordingly, the present work should be interpreted as an integrative narrative review rather than as a systematic review.

## 3. Central Sensitization and Altered Nociceptive Gain

Central sensitization represents one of the principal mechanisms proposed to underlie pain amplification in fibromyalgia [1,10,11,12]. Its relevance is supported by the consistent observation that patients exhibit increased pain sensitivity across several stimulus modalities, including pressure, heat, cold, and electrical stimulation [6,10,16]. Importantly, hypersensitivity is not confined to classical tender points or to the sites of greatest spontaneous pain, but extends more diffusely across the body, supporting an alteration in nociceptive gain rather than a purely localized musculoskeletal abnormality [5,6,10,11].

Among the most informative experimental findings is enhanced temporal summation of pain. Repetitive nociceptive stimulation can progressively increase perceived pain, providing a psychophysical correlate of activity-dependent amplification within central nociceptive pathways [12,24,25,26]. Patients with fibromyalgia have repeatedly shown greater temporal summation of second pain, more pronounced painful after-sensations, and slower decay of pain following repeated stimulation than healthy controls [11,24,25,26,27]. These observations are consistent with increased excitability and persistence of nociceptive processing and provide experimental support for altered central pain amplification in fibromyalgia [12,24,25,26,27].

The interpretation of these findings nevertheless requires caution. Temporal summation, pain thresholds, and other quantitative sensory testing measures are indirect experimental correlates of central nociceptive processing rather than direct measurements of the cellular phenomenon of central sensitization itself [11,12,16]. Increased pain sensitivity therefore supports altered nociceptive gain but should not automatically be regarded as evidence that an identical neurophysiological mechanism is operating to the same extent in every patient [11,12,16]. This distinction is particularly relevant in fibromyalgia, where experimental sensory profiles show substantial inter-individual variability [11,15,16].

At the mechanistic level, activity-dependent facilitation of nociceptive transmission involves enhanced excitatory signaling, including glutamatergic mechanisms and NMDA receptor-dependent plasticity [10,12]. Such processes can increase the responsiveness of spinal nociceptive neurons and promote amplification that persists beyond the initiating input [12]. In fibromyalgia, neurochemical and neuroimaging findings are broadly compatible with this model, including altered glutamatergic signaling [28,29] and augmented or reorganized activity within regions involved in pain processing, including the insula, anterior cingulate cortex, thalamus, and prefrontal cortex [1,6,11,30]. These findings indicate that altered nociceptive gain is unlikely to be restricted to the spinal cord but instead involves distributed central networks contributing to sensory-discriminative, affective, and cognitive dimensions of pain [1,6,11,30].

This broader network perspective may also help to account for the frequent coexistence of pain hypersensitivity with enhanced responsiveness to non-nociceptive sensory stimuli, including sound, light, and odors [1,2,4]. Such manifestations are compatible with a more generalized disturbance of sensory processing, although they should not be assumed to arise exclusively from the same mechanisms responsible for nociceptive amplification [1,2,4]. Central sensitization is therefore best considered within a distributed sensory-regulatory framework rather than as an isolated spinal phenomenon [1,11,12].

Importantly, however, central sensitization is neither uniformly expressed nor sufficient to explain the entire fibromyalgia phenotype [1,11,16]. Although group-level evidence consistently supports pain hypersensitivity, abnormalities in specific experimental indices are less uniform [11,15,16]. Some studies have demonstrated clear hyperalgesia without marked abnormalities in every measure of temporal summation or endogenous pain modulation [11,15]. Accordingly, central amplification appears to vary in magnitude and expression across patients rather than constituting a single invariant physiological signature of fibromyalgia [11,15,16].

Taken together, the psychophysical, neurochemical, and neuroimaging evidence supports altered central nociceptive gain as a major feature of fibromyalgia, with enhanced temporal summation and prolonged pain responses providing particularly relevant experimental correlates [1,10,11,12,24,25,26,28,29,30,31]. At the same time, variability across patients and paradigms argues against equating fibromyalgia with a single central sensitization phenotype [11,15,16].

## 4. Descending Pain Modulation in Fibromyalgia

Descending pain modulation represents a major determinant of nociceptive gain and is frequently altered in fibromyalgia [1,11,15,31,32]. Rather than functioning as a purely inhibitory system, descending control is intrinsically bidirectional: supraspinal and brainstem networks can suppress or facilitate nociceptive transmission according to behavioral context, ongoing sensory input, and physiological state [33,34,35,36,37,38]. The net pain experience therefore reflects not only the strength of ascending nociceptive signaling but also the dynamic balance between descending inhibitory and facilitatory influences [33,35,38].

Key components of this system include cortical and subcortical regions such as the prefrontal and anterior cingulate cortices, together with brainstem structures including the periaqueductal gray (PAG), rostral ventromedial medulla (RVM), locus coeruleus (LC), raphe nuclei, and dorsal reticular nucleus (DRt/SRD) [33,34,35,36,37,38]. These structures form interacting networks rather than a single linear pathway [33,35,38]. Experimental studies have established that the RVM contains functionally distinct neuronal populations capable of exerting inhibitory or facilitatory effects on spinal nociceptive processing [33,35], whereas the DRt represents an important pronociceptive component of descending control [34,36,37]. Noradrenergic projections from the LC and related pontine nuclei provide an additional state-dependent modulatory influence on spinal nociceptive processing [38]. Although much of this circuit-level knowledge derives from preclinical studies, it provides an essential mechanistic framework for interpreting abnormalities of endogenous pain control in humans [33,34,35,36,37,38].

Conditioned pain modulation (CPM) is the most widely used psychophysical paradigm for assessing endogenous pain regulation in humans and is conceptually related to diffuse noxious inhibitory controls (DNICs) [39,40,41]. In a typical CPM paradigm, a conditioning noxious stimulus alters the perceived intensity of a second test stimulus applied at a remote site [40,41]. Although hypoalgesia is the expected response in many healthy individuals, CPM responses show substantial inter-individual variability and may include weak inhibition, no change, or even facilitation [15,40,41,42]. CPM should therefore be interpreted as the net behavioral expression of interacting facilitatory and inhibitory processes rather than as a direct or selective measure of a single descending inhibitory pathway [15,35,40,41].

At the group level, a substantial literature supports reduced CPM efficacy in fibromyalgia [15,31,32,43]. Earlier experimental studies reported deficient endogenous pain inhibition [31,43], and a meta-analysis of temporal summation and CPM paradigms confirmed a significant group-level impairment while also identifying considerable methodological heterogeneity across studies [31,32,41,43]. These findings support altered endogenous pain control in fibromyalgia, but the magnitude and even the direction of the abnormality vary across individuals and experimental paradigms [11,15,32,41]. Thus, impaired CPM should not be regarded as a universal trait of the syndrome.

Particularly relevant to this heterogeneity, Potvin and Marchand demonstrated marked inter-individual variability in CPM responses in fibromyalgia, including patients in whom conditioning stimulation produced pain facilitation rather than inhibition [15]. This observation is mechanistically important because an abnormal CPM response cannot always be understood simply as absent analgesia [15,41]. In some patients, the net balance of endogenous modulation may shift toward facilitation, such that conditioning stimulation increases rather than decreases test pain [15,33,35,41,42]. Such responses are consistent with the broader concept that chronic pain may involve altered weighting of inhibitory and facilitatory control [33,35].

At the same time, studies using test stimuli individually adjusted to account for baseline hypersensitivity have confirmed pronounced mechanical and thermal hyperalgesia in fibromyalgia while failing to demonstrate consistent abnormalities of temporal summation or descending pain inhibition [11]. In related spinal and brainstem fMRI experiments, patients showed broadly similar temporal patterns of spinal activation but increased brainstem BOLD activity during pain summation despite comparable behavioral modulation [11]. These findings indicate that hypersensitivity and abnormal endogenous pain modulation are related but separable phenomena and caution against assuming that impaired CPM or enhanced temporal summation is demonstrable in every patient or under every experimental condition [11].

Human neuroimaging further supports altered organization of descending modulatory networks. Resting-state fMRI studies have demonstrated abnormal PAG functional connectivity in fibromyalgia, including altered coupling with cortical regions involved in pain, affective processing, and endogenous modulation [44,45]. Soldatelli et al. further showed that fibromyalgia patients classified as CPM non-responders had reduced functional connectivity between the primary somatosensory cortex and the PAG compared with CPM responders [46]. These findings link individual differences in behavioral pain modulation to variation in central network organization [44,45,46]. Nevertheless, functional connectivity and BOLD signals do not directly identify inhibitory or facilitatory neuronal output, and these imaging abnormalities cannot by themselves determine whether a given pattern reflects reduced inhibition, increased facilitation, compensatory recruitment, or a consequence of persistent pain [44,45,46].

Recent ultra-high-field human imaging provides an additional framework for interpreting this issue. Wake et al. used 7-T fMRI during CPM in pain-free individuals and demonstrated that conditioning-induced hypoalgesia and hyperalgesia were associated with partly dissociable patterns of brainstem activity involving the PAG, RVM, LC, A5 region, and subnucleus reticularis dorsalis (SRD) [42]. Across individuals, changes in pain were also related to activity within PAG and RVM regions, reinforcing the concept that human CPM recruits a distributed brainstem network capable of supporting both pain reduction and pain enhancement [42]. Importantly, this study was conducted in pain-free participants and should therefore not be interpreted as direct evidence that an equivalent facilitatory brainstem configuration is specifically enhanced in fibromyalgia [42]. Rather, it provides a human physiological framework within which facilitatory CPM responses observed in subsets of patients with fibromyalgia can be interpreted [15,42].

The distinction between behavioral output and circuit mechanism is critical. Pain facilitation during CPM demonstrates a shift in the net modulatory response but does not identify a single underlying descending pathway [15,33,35,41,42]. Increased net facilitation could theoretically involve altered RVM output, recruitment of pronociceptive reticular circuitry such as the DRt/SRD, changes in monoaminergic modulation, altered cortical–brainstem coupling, or combinations of these mechanisms [33,34,35,36,37,38,44,45,46]. However, direct pathway-specific demonstration of enhanced descending facilitation in patients with fibromyalgia remains limited. Mechanistic interpretation should therefore distinguish between established human behavioral observations and circuit mechanisms inferred from experimental and neuroimaging evidence [15,33,34,35,36,37,38,42,44,45,46].

Neurochemical alterations may further influence this balance. Serotonergic, noradrenergic, opioidergic, and dopaminergic systems participate in descending pain regulation [35,38,47,48,49,50,51], and abnormalities involving these systems have been described in fibromyalgia [2,4,48,50,51]. Such findings provide biological plausibility for altered descending modulation but do not demonstrate that deficiency of any single neurotransmitter system independently causes the disorder [4,35,38,48,49,50,51]. The relevant neurochemical abnormalities and their interaction with excitatory pain amplification are considered in greater detail in the following section.

Overall, reduced CPM efficacy is supported at the group level, yet individual responses range from effective inhibition to absent inhibition and, in some patients, pain facilitation [15,31,32,42,43]. Differences produced by stimulus calibration and experimental paradigm further indicate that descending dysfunction is context- and paradigm-dependent rather than an invariant physiological trait [11,32,41]. A more accurate interpretation is not that fibromyalgia reflects a universal loss of endogenous analgesia, but that the normal weighting of inhibitory and facilitatory control is variably reconfigured across individuals [11,15,32,33,35,41]. This altered balance may interact with enhanced ascending nociceptive gain to sustain pain amplification while also contributing to the mechanistic heterogeneity of the syndrome [1,11,15,35].

## 5. Neurochemical Dysregulation

Neurochemical alterations provide an important molecular substrate for abnormal nociceptive gain in fibromyalgia, although the syndrome cannot be attributed to dysfunction of any single neurotransmitter system [1,2,4,6]. The most consistent findings point to alterations in the balance between excitatory signaling and endogenous inhibitory regulation, particularly involving glutamatergic transmission, substance P, monoaminergic pathways, endogenous opioid signaling, and γ-aminobutyric acid (GABA) [2,4,6,10,28,29,48,50,51,52,53,54].

Glutamate is particularly relevant because of its central role in activity-dependent synaptic plasticity and central sensitization [10,12]. Proton magnetic resonance spectroscopy studies have demonstrated increased glutamatergic measures in pain-related regions in fibromyalgia, particularly the insula, and higher insular glutamate has been associated with greater experimental pain sensitivity [28,29]. Changes in insular glutamate accompanying clinical improvement have also been reported [28]. Collectively, these observations support a relationship between glutamatergic signaling and pain processing in fibromyalgia, while not establishing increased glutamate as a primary causal abnormality [28,29].

At the spinal level, glutamate acts through both ionotropic and metabotropic receptors involved in activity-dependent nociceptive plasticity. NMDA receptor activation is particularly important for increases in synaptic efficacy and calcium-dependent intracellular signaling associated with central sensitization and long-term potentiation (LTP)-like changes in dorsal horn nociceptive transmission [12,55]. Experimental spinal studies further demonstrate that induction of LTP of C-fiber-evoked responses requires recruitment of group I metabotropic glutamate receptors (mGluRs), particularly mGluR1/5, whereas group II/III mGluRs do not appear to play the same requisite role in this form of potentiation [55]. Thus, NMDA receptors and group I mGluRs participate in related but non-equivalent components of activity-dependent spinal plasticity: NMDA receptors provide a major ionotropic mechanism for induction of potentiation, whereas group I mGluRs engage G-protein-dependent signaling that can facilitate the development of sustained synaptic enhancement [12,55].

These spinal mechanisms provide a plausible framework for the enhanced temporal summation and persistent nociceptive gain observed in fibromyalgia [11,12,24,25]. However, an important distinction is required: NMDA- and mGluR-dependent LTP has been characterized predominantly in experimental models of nociceptive plasticity, whereas direct evidence for disease-specific mGluR1/5 dysfunction in patients with fibromyalgia remains limited [55]. The experimental literature therefore provides a mechanistic framework for interpreting amplified nociceptive processing rather than evidence that an mGluR abnormality is itself a defining molecular lesion of fibromyalgia.

Pharmacological observations provide additional, but indirect, support for NMDA receptor involvement. In an early double-blind, placebo-controlled study, intravenous ketamine produced short-term reductions in pain intensity and tenderness in a subgroup of patients with fibromyalgia [56]. Such findings are consistent with involvement of NMDA-dependent signaling in pain maintenance, but pharmacological responsiveness does not demonstrate that abnormal NMDA receptor function is a primary cause of fibromyalgia [12,56]. Ketamine is therefore better regarded as a mechanistic probe supporting the relevance of NMDA-dependent nociceptive amplification than as evidence for a disease-specific receptor abnormality.

Substance P represents another well-established pronociceptive neurochemical finding. Cerebrospinal fluid concentrations have been reported to be approximately threefold higher in patients with fibromyalgia than in healthy controls [52]. Through neurokinin-1 (NK1) receptors, substance P can facilitate nociceptive transmission and interact with glutamatergic mechanisms, thereby favoring spinal and supraspinal excitability [3,6,10,52]. However, elevated substance P does not by itself explain the fibromyalgia phenotype and is more appropriately interpreted as one component of a broader pronociceptive neurochemical environment than as an independent or disease-specific biomarker [6,10,52].

In parallel with excitatory abnormalities, altered monoaminergic regulation has long been described in fibromyalgia [4,6,48]. Russell et al. reported reduced cerebrospinal fluid concentrations of metabolites of serotonin, noradrenaline, and dopamine in patients with fibromyalgia compared with controls [48]. Because serotonin and noradrenaline contribute to descending modulation of nociceptive transmission, these findings are compatible with altered endogenous pain regulation [47,48,49]. Their interpretation nevertheless requires caution: concentrations of metabolites in cerebrospinal fluid provide an indirect index of neurotransmitter turnover and do not measure transmitter release or receptor signaling within specific descending pathways [47,48]. They therefore cannot establish that a primary monoaminergic deficiency causes fibromyalgia.

The partial clinical efficacy of serotonin–noradrenaline reuptake inhibitors and other monoamine-modulating drugs is likewise compatible with the biological relevance of these systems [4,6]. Therapeutic response, however, demonstrates that modifying monoaminergic transmission can influence symptoms; it does not establish that deficient serotonin or noradrenaline transmission constitutes the initiating pathogenic abnormality. Monoaminergic dysfunction may be primary in some contexts, secondary to persistent pain or stress-system alterations, or part of a broader compensatory reorganization of pain-regulatory networks [4,6,47,48,49].

Dopaminergic signaling adds another dimension to this profile. Dopamine participates in supraspinal pain modulation as well as reward, motivation, motor control, and cognitive-affective processing [4,6,50]. PET studies have demonstrated an abnormal dopaminergic response to experimental pain in patients with fibromyalgia [50]. Together with the reduced dopamine metabolite concentrations reported in cerebrospinal fluid [48], these observations support altered dopaminergic regulation rather than a simple state of dopamine deficiency [48,50]. The functional consequences may extend beyond nociception, although direct causal links between dopaminergic abnormalities and individual non-pain symptoms remain insufficiently established.

The endogenous opioid system also appears to be dysregulated, but available evidence argues against a simple opioid-deficiency model [6,51]. PET imaging has demonstrated reduced central μ-opioid receptor availability in fibromyalgia, including in regions involved in pain and affective processing [51]. This finding may reflect altered receptor expression, increased occupancy by endogenous ligands, or changes in receptor regulation rather than reduced endogenous opioid release per se [6,51]. Indeed, earlier measurements of cerebrospinal fluid β-endorphin did not demonstrate a straightforward reduction in patients with fibromyalgia. Thus, opioid dysfunction is better conceptualized as altered endogenous opioid signaling and receptor dynamics than as an absolute deficiency of endogenous opioid activity [6,51].

Alterations in inhibitory neurotransmission have also been identified. Proton magnetic resonance spectroscopy has demonstrated reduced GABA concentrations in the anterior insula of patients with fibromyalgia [53], whereas PET imaging has shown increased cortical GABA-A receptor concentration in several regions [54]. These apparently different findings illustrate that abnormalities in inhibitory signaling cannot be reduced to a uniform global loss of GABAergic function [53,54]. Together with the glutamatergic findings [35,36], they instead suggest regionally specific alterations in the balance between excitation and inhibition within pain-processing networks [28,29,53,54].

Overall, the neurochemical evidence supports altered excitatory–inhibitory and modulatory balance rather than a single transmitter defect [2,4,6]. Increased glutamatergic signaling and substance P, altered monoaminergic and dopaminergic regulation, dysregulated endogenous opioid signaling, and region-specific changes in GABAergic function may each contribute to increased nociceptive gain or altered endogenous pain control [28,29,48,49,51,52,53,54]. However, these findings differ substantially in anatomical level, methodology, and evidential strength, and most support association or mechanistic plausibility rather than a simple linear causal sequence [4,6,28,29,48]. Neurochemical dysregulation should therefore be understood as one interacting component of the broader fibromyalgia phenotype, whose expression and relative importance are likely to vary across patients and disease states.

## 6. Peripheral Nociceptive Contributors

Although fibromyalgia is characterized by prominent abnormalities of central pain processing, peripheral nociceptive input may contribute meaningfully to the initiation, amplification, or persistence of pain in at least a subset of patients [6,10,17,20,21,57]. Peripheral and central mechanisms should not be regarded as competing explanations. Persistent afferent input can promote activity-dependent changes within the spinal cord and brain, while altered central processing can, in turn, amplify the perceptual consequences of relatively modest peripheral signals [6,10,20,27,57].

Deep-tissue nociception is particularly relevant because spontaneous pain in fibromyalgia is commonly perceived in muscles and other deep somatic structures rather than being predominantly cutaneous [10,57]. Patients also show enhanced sensitivity to pressure and other forms of deep-tissue stimulation, and exaggerated temporal summation has been demonstrated using mechanical stimulation of muscle tissue [10,27,57]. These observations suggest that muscle and related deep tissues may contribute to the afferent drive entering an already sensitized nociceptive system [6,10,27,57]. Such a contribution does not imply a primary structural muscle disorder; relatively subtle metabolic, biochemical, or microcirculatory abnormalities could modify the excitability of muscle nociceptors and thereby influence central nociceptive gain [10,57,58,59,60].

Human microdialysis studies provide direct evidence of altered local muscle biochemistry in fibromyalgia. Gerdle et al. reported increased interstitial lactate and pyruvate concentrations in resting trapezius muscle in women with fibromyalgia compared with healthy controls [58]. In a subsequent study of the vastus lateralis, women with fibromyalgia showed increased interstitial glutamate and pyruvate concentrations before exercise training, with these abnormalities becoming less pronounced after a structured exercise intervention [59]. Combined microdialysis and phosphorus-31 magnetic resonance spectroscopy further showed higher pyruvate concentrations, reduced phosphocreatine and nucleotide triphosphate levels—predominantly reflecting ATP—and reduced muscle blood flow, with some metabolic variables associated with pain intensity and pressure-pain thresholds [60]. Collectively, these findings support measurable peripheral biochemical and metabolic alterations in at least some muscles of patients with fibromyalgia. However, causal direction remains uncertain, with possible contributions from altered muscle use, deconditioning, inactivity, obesity, aging, and chronic pain itself [58,59,60]. The evidence therefore supports a peripheral metabolic component, but does not demonstrate that these abnormalities constitute a primary mitochondrial or muscular etiology of fibromyalgia.

The nociceptive relevance of such metabolic changes is biologically plausible. Skeletal-muscle afferents, particularly group III and IV fibers, are sensitive to changes in the local chemical environment generated during contraction, ischemia, or metabolic stress [61]. Experimental studies have shown that combinations of protons, ATP, and lactate activate muscle-innervating dorsal root ganglion neurons more effectively than the same mediators administered individually, indicating that metabolite interactions may be particularly important in muscle nociception [61]. This response involves coordinated signaling through acid-sensing ion channels (ASICs), purinergic P2X receptors, and TRPV1-related mechanisms [61]. Two functionally distinguishable populations of muscle afferents have also been described: lower metabolite concentrations preferentially activate metaboreceptive pathways, whereas higher and more noxious concentrations recruit metabo-nociceptive pathways [61].

These experimental findings provide a mechanistic framework through which altered muscle metabolism could contribute to pain amplification in fibromyalgia. Increased extracellular acidity and concentrations of metabolic products may lower nociceptor activation thresholds, enhance peripheral afferent discharge, and thereby increase nociceptive input to the spinal cord [57,61]. However, the evidentiary basis requires careful separation. Increased lactate, pyruvate, glutamate, and alterations in muscle energy metabolism have been reported in human fibromyalgia studies [58,59,60], whereas the precise contribution of individual ASIC, P2X, and TRPV1-related mechanisms to fibromyalgia pain remains largely inferred from experimental studies of muscle-nociceptor physiology [61]. Thus, these data support biological plausibility for a peripheral contribution to nociceptive amplification, but do not establish that dysfunction of any individual metabolite-sensitive receptor system is a demonstrated pathogenic mechanism in fibromyalgia.

Peripheral sensitization may extend beyond metabolic mechanisms. Altered tissue perfusion, neuropeptides, inflammatory mediators, and other locally released biochemical signals can increase the excitability of peripheral nociceptors and enhance afferent signaling [3,10,20,57]. However, tissue-level findings in fibromyalgia are heterogeneous and have not established a uniform peripheral lesion. Peripheral mechanisms should therefore be regarded as potential sources or amplifiers of nociceptive input rather than as a single defining pathology [6,10,57].

The contribution of ongoing peripheral input may be particularly apparent when fibromyalgia coexists with another painful condition. Patients with inflammatory rheumatic disease, osteoarthritis, or other persistent nociceptive disorders may develop a fibromyalgia phenotype in which widespread hypersensitivity and central amplification coexist with a continuing peripheral nociceptive source [6,17,18,19,20]. This observation supports the concept that similar clinical manifestations may arise from different relative combinations of central amplification and peripheral drive [6,17,18,19,20].

This interaction is better represented as a continuum than as a dichotomy [6,17,18,19,20]. In some patients, central amplification may dominate the clinical phenotype despite limited identifiable peripheral input; in others, persistent deep-tissue nociception or another peripheral pain generator may contribute more substantially [6,10,17,18,19,57]. The relative weighting of these mechanisms may also change over time, allowing peripheral input to function as an initiating factor, an amplifier, or a recurrent trigger without remaining the sole driver of chronic widespread pain [6,10,57].

Overall, peripheral nociceptive mechanisms should be viewed as variable components of the fibromyalgia pain system rather than as either irrelevant epiphenomena or universal primary causes [6,17,18,19,20,57]. Deep-tissue metabolic abnormalities and peripheral sensitization may provide afferent input capable of interacting with central sensitization and altered descending modulation [6,10,27,57,58,59,60,61], while the magnitude and clinical relevance of this contribution are likely to differ across patients. This framework provides an important transition to one of the most intensively investigated peripheral findings in fibromyalgia: small-fiber pathology, whose role as a primary driver, phenotypic marker, or downstream consequence remains unresolved [19,21].

## 7. Small Fiber Pathology: Peripheral Driver, Phenotypic Marker, or Downstream Consequence?

Small-fiber pathology (SFP) has emerged as one of the most important and debated peripheral findings in fibromyalgia. Reduced intraepidermal nerve fiber density (IENFD), abnormalities of small-fiber morphology and function, and corneal nerve changes have been demonstrated in a substantial subgroup of patients [62,63,64,65,66,67]. Meta-analytic evidence suggests that approximately one-half of patients may show evidence of small-fiber pathology, although prevalence estimates vary according to methodology and diagnostic thresholds [19,63]. These findings establish that peripheral small-fiber abnormalities are common in fibromyalgia, but their pathogenic significance remains unresolved [19,21,63].

A key distinction is that SFP in fibromyalgia should not be equated with classical small-fiber neuropathy (SFN) [19,68,69,70]. Although both conditions may involve reduced IENFD and overlapping symptoms such as burning pain, paresthesia, dysesthesia, and autonomic complaints, their clinical, functional, and morphological profiles differ [19,68,69,70,71]. Patients with fibromyalgia-related SFP may lack the clear negative sensory signs and thermal detection abnormalities characteristic of established SFN, and the distribution of fiber loss is frequently non-length-dependent [19,70,72,73]. Abnormalities of cold and warm detection thresholds have not been consistently replicated across studies and should not be interpreted as a uniform small-fiber sensory signature; the most consistently reported quantitative sensory findings relate to pain hypersensitivity and features compatible with central amplification [19,70,72]. Recent comparative evidence further indicates that IENFD reduction is quantitatively milder in fibromyalgia-related SFP than in SFN at both distal and proximal biopsy sites [73]. SFPs associated with fibromyalgia and classical SFN are therefore related but distinct peripheral phenotypes rather than interchangeable diagnoses [19,68,69,73].

Reduced IENFD alone is insufficient to establish SFN in a patient with fibromyalgia; skin-biopsy findings must be interpreted alongside the clinical phenotype, neurological examination, sensory testing, and the anatomical pattern of fiber loss [68,69]. Conversely, true SFN may coexist with fibromyalgia and should be considered when the presentation includes objective sensory deficits, abnormal thermal detection, or a degree and distribution of fiber loss more typical of peripheral neuropathy [16,68,69,70,73]. Reduced IENFD should not in itself lead to reclassification of fibromyalgia as a peripheral neuropathy.

The biological meaning of SFP in fibromyalgia remains more difficult to define. One possibility is that reduced small-fiber innervation identifies a subgroup in which peripheral nociceptive abnormalities contribute more strongly to ongoing afferent input [19,21,70]. Small fibers include nociceptive Aδ- and C-fibers as well as autonomic fibers, and their dysfunction may therefore influence both pain and autonomic regulation [68,71,74]. In this model, structural or functional abnormalities of peripheral afferents could provide sustained input capable of reinforcing central sensitization and altered nociceptive gain [19,21].

However, available evidence does not support a simple model in which peripheral axonal degeneration constitutes the primary cause of fibromyalgia. Reduced IENFD does not correlate consistently with somatosensory dysfunction or symptom severity, and clinically well-characterized patients with fibromyalgia may show marked pain hypersensitivity despite little or no demonstrable small-fiber impairment [16,19,72]. Fasolino et al., for example, found that the presence of SFP had no major impact on somatosensory system function in patients with fibromyalgia [72]. The quantitatively milder IENFD reduction recently demonstrated in fibromyalgia-related SFP compared with classical SFN further suggests that peripheral fiber loss alone is unlikely to account for widespread pain and the broader multisystem phenotype [73].

An alternative possibility is that SFP may arise downstream of broader nociceptive or neurobiological dysregulation. In a proof-of-concept study, Harte et al. reported reduced intraepidermal nerve fiber density following a sustained increase in insular glutamate, raising the possibility that central excitatory dysregulation may contribute to peripheral small-fiber changes rather than necessarily resulting from them [75]. Although this finding does not establish a general causal pathway in fibromyalgia, it challenges a strictly bottom-up interpretation of SFP and illustrates the potential bidirectionality of central–peripheral interactions.

A further mechanistic hypothesis has emerged from neuroimmune studies focused on the dorsal root ganglia (DRG). Passive transfer of immunoglobulin G (IgG) from patients with fibromyalgia to mice produced mechanical and cold hypersensitivity, increased responsiveness of peripheral nociceptive afferents, and reduced intraepidermal innervation [76]. Patient IgG accumulated within the DRG and showed prominent binding to satellite glial cells surrounding sensory neurons [76]. These observations raise the possibility that, in at least a subset of patients, peripheral nociceptor dysfunction and small-fiber loss may arise from immune-mediated processes centered on the sensory ganglia rather than from a conventional distal axonopathy [20,76].

This hypothesis is strengthened by subsequent human evidence demonstrating elevated IgG binding to satellite glial cells in subsets of patients with fibromyalgia [77]. Higher anti-SGC IgG levels were associated with greater pain intensity, increased pressure sensitivity, and greater overall fibromyalgia severity [77]. Together with the passive-transfer findings [76], these observations suggest a potential DRG–satellite glial cell–nociceptor axis in which immune signaling alters the excitability and local environment of primary sensory neurons. Importantly, this remains an emerging mechanistic model rather than an established explanation for SFP across the fibromyalgia population [20,76,77].

Small-fiber abnormalities may therefore have several non-mutually exclusive interpretations. They may contribute directly to nociceptive input in some patients, identify a phenotype with greater peripheral involvement, reflect secondary changes driven by central excitatory or neuroimmune mechanisms, or coexist with fibromyalgia as a partially independent process [19,21,70,75,76]. The relative contribution of these possibilities is likely to differ between individuals.

Phenotyping studies support this view. Patients with fibromyalgia and reduced small-fiber innervation may show sensory profiles that differ from those without measurable SFP, although the separation is incomplete [16,19,66,70]. Leone et al. identified distinct sensory phenotypes associated with small-fiber abnormalities in fibromyalgia and SFN [70], while corneal nerve fiber quantification has also been used to derive peripheral phenotypes in fibromyalgia [66,67]. Skin biopsy, corneal confocal microscopy, QST, and autonomic testing may therefore provide complementary phenotypic information, but no single peripheral measure currently defines a prospectively validated fibromyalgia subtype [19,66,70].

Autonomic small fibers deserve particular consideration. Skin-biopsy studies have demonstrated reduced autonomic innervation of sweat glands and piloerector muscles in patients with fibromyalgia-associated SFP [74]. Importantly, however, the degree of autonomic small-fiber loss did not correlate with the severity of self-reported autonomic symptoms [74]. This dissociation suggests that peripheral autonomic SFP may contribute to dysautonomia in some patients but cannot by itself account for the broader autonomic phenotype, which likely also involves central autonomic regulation. This distinction becomes particularly relevant in the discussion of autonomic dysfunction later in the review.

Overall, SFP in fibromyalgia is best regarded as a reproducible but heterogeneous biological finding whose mechanistic meaning remains context-dependent [19,21,63,70]. It differs clinically and quantitatively from classical SFN [68,70,73], is not present in all patients [19,63], and does not by itself account for the full fibromyalgia phenotype [19,72]. In some individuals, SFP may contribute to ongoing peripheral nociceptive input; in others, it may represent a downstream consequence of central excitatory, neuroimmune, or DRG-related processes [75,76]. This multidirectional interpretation avoids a false choice between purely central and purely peripheral models and provides a natural bridge to the broader neuroimmune mechanisms considered in the following section.

## 8. Neuroinflammation and Neuroimmune Mechanisms

Neuroimmune signaling has become an increasingly important component of mechanistic models of fibromyalgia [1,20,78,79]. Rather than implying a classical systemic inflammatory disorder, the available evidence suggests alterations in communication among immune cells, glial cells, primary sensory neurons, and central pain-processing networks that may influence nociceptive gain in at least a subset of patients [20,78,79]. These processes may operate at multiple anatomical levels, including peripheral tissues, dorsal root ganglia (DRG), spinal nociceptive circuits, and supraspinal networks [3,20,78].

One of the most prominent lines of evidence concerns glial-associated neuroinflammatory signaling in the brain. Positron emission tomography studies using ligands targeting the 18-kDa translocator protein (TSPO) have demonstrated increased binding in several cortical regions in patients with fibromyalgia, including frontal, parietal, cingulate, somatosensory, and motor areas [20,79,80]. In the multi-site study by Albrecht et al., increased [^11C]PBR28 signal was widespread and, in some regions, associated with greater fatigue [80]. These findings provide in vivo evidence of altered TSPO-associated neuroimmune activity [78,79,80], but not direct proof of microglial activation, because TSPO is expressed by microglia, astrocytes, endothelial cells, and other vascular-associated cells. The absence of increased astrocyte-enriched [^11C]-L-deprenyl-D2 binding in the fibromyalgia PET study may suggest a relatively greater microglial contribution, but this remains an indirect inference [80]. TSPO PET should therefore be interpreted as evidence of altered neuroimmune or glial-associated activity rather than as a selective marker of microglial activation [80,81].

Peripheral and systemic immune findings provide a complementary, although heterogeneous, line of evidence. Studies have reported alterations in circulating cytokines and chemokines, including IL-6, IL-8, TNF-related signaling, and other inflammatory mediators, but results vary considerably across cohorts and biological samples [1,4,20,79]. Meta-analytic and review-level evidence supports modest immune dysregulation rather than a uniform systemic inflammatory state [4,20,79], and differences in adiposity, physical activity, sleep, medication, psychiatric comorbidity, and coexisting inflammatory conditions may contribute to this variability [20,79]. The functional relevance of such immune signals lies in their ability to modify nociceptive processing at several levels. Cytokines and related mediators can alter peripheral nociceptor excitability, influence glial and neuronal signaling within the spinal cord, and modify synaptic plasticity and descending pain regulation [1,20,78,79]. These mechanisms therefore provide plausible routes through which immune activation could amplify pain without requiring overt tissue inflammation or a conventional inflammatory disease [20,78].

The DRG has emerged as a particularly important neuroimmune interface [3,20]. Sensory neuron cell bodies are closely surrounded by satellite glial cells (SGCs), which regulate neuronal homeostasis and can participate in inflammatory and nociceptive signaling [20,76]. In this context, immune-mediated changes can modify nociceptor excitability without necessarily producing a classical distal peripheral neuropathy. This provides an important mechanistic bridge between the peripheral small-fiber abnormalities discussed in the preceding section and broader neuroimmune processes [19,20,76].

A major development in this field came from passive-transfer experiments demonstrating that immunoglobulin G (IgG) from patients with fibromyalgia induces pain-like hypersensitivity in mice [76]. Fibromyalgia IgG accumulated in the DRG, bound to satellite glial cells and other sensory-ganglion structures, increased nociceptor responsiveness, and was accompanied by reduced intraepidermal innervation [76]. These findings provide experimental evidence that circulating IgG from at least some patients can exert pronociceptive effects independently of generalized inflammation [76].

Subsequent human studies have strengthened this observation. Elevated anti-SGC IgG has been identified in subsets of patients with fibromyalgia and has been associated with greater pain intensity, pressure sensitivity, and overall symptom severity [77]. More recent studies have also linked anti-SGC IgG levels to altered lipid profiles and pain-related metabolic signatures, suggesting that autoreactive immune mechanisms may interact with nociceptor membrane biology and cellular signaling [82]. Importantly, these associations do not establish that anti-SGC antibodies are present or pathogenic in all patients.

Additional evidence further supports heterogeneity in DRG-directed immune reactivity. In a recent cohort study, IgG binding to DRG structures was detected in a substantial subset of patients with fibromyalgia but not in control sera, with distinct binding patterns involving neurons and satellite glial cell-associated structures [83]. Specific patterns were associated with clinical features such as pain intensity and burning pain [83]. These observations reinforce the possibility that immune-mediated sensory-ganglion mechanisms contribute to defined fibromyalgia phenotypes rather than constituting a universal disease mechanism.

The emerging IgG literature therefore raises an important conceptual possibility: small-fiber pathology, nociceptor sensitization, and pain amplification may in some patients represent parallel consequences of an upstream DRG-centered neuroimmune process [19,20,76,77,83]. In such a model, immune interactions with satellite glial cells or sensory neurons could increase peripheral afferent activity and alter the trophic environment of small fibers, while persistent nociceptive input subsequently reinforces central sensitization and altered descending modulation [20,76]. This model remains provisional but provides a mechanistically coherent connection between peripheral, immune, and central findings.

These observations should not be interpreted as establishing fibromyalgia as a conventional autoimmune disease [4,20]. No single autoantibody has been shown to define the syndrome, immune abnormalities are not consistently present across patients, and the relevant antigenic targets and pathogenic pathways remain incompletely characterized [20,76,83]. A more appropriate interpretation is that autoreactive IgG and related neuroimmune mechanisms may contribute to pain in biologically distinct subsets of patients.

Central and peripheral neuroimmune processes may also interact bidirectionally [20,78,79]. Persistent nociceptive activity can alter glial function and immune signaling, while inflammatory mediators can increase neuronal excitability and synaptic gain [78,79]. Stress-system activity, autonomic regulation, sleep disturbance, and metabolic factors may further modify this interface [1,4,20]. Neuroimmune abnormalities may therefore function as both drivers and consequences of chronic pain-related dysregulation, making causal direction difficult to infer from cross-sectional studies.

Overall, current evidence supports neuroimmune dysregulation as a biologically relevant but heterogeneous component of fibromyalgia [1,20,78,79]. TSPO PET provides evidence of altered central neuroimmune activity but not cell-specific proof of microglial activation [80,81], while emerging IgG and DRG–satellite glial cell findings provide increasingly compelling evidence for peripheral neuroimmune mechanisms in subsets of patients [76,77,83]. Rather than constituting a single inflammatory explanation for fibromyalgia, these mechanisms are best understood as interacting components capable of modifying peripheral nociceptor function, small-fiber integrity, central nociceptive gain, and symptom severity. Their relative contribution is likely to vary across patients and may ultimately prove useful for mechanism-based phenotyping.

## 9. Stress-System, Autonomic, Sleep, and Endocrine Modulation

Stress-regulatory, autonomic, sleep, and endocrine alterations are best viewed as interacting modulators rather than independent or stereotyped causes of fibromyalgia [4,6,20,84,85,86,87,88]. These systems influence nociceptive processing, arousal, fatigue, sleep continuity, cardiovascular regulation, and neuroimmune signaling and may therefore modify both pain severity and the broader symptom burden [1,4,6,20]. Importantly, abnormalities within these domains are heterogeneous and often state-dependent, arguing against a single stereotyped pattern of stress-system dysfunction across patients.

### 9.1. The Hypothalamic–Pituitary–Adrenal (HPA) Axis and Stress Reactivity

The hypothalamic–pituitary–adrenal (HPA) axis has long been investigated in fibromyalgia because of its central role in coordinating physiological responses to stress [84,85,88]. Early studies described altered basal cortisol concentrations, abnormal diurnal profiles, impaired dexamethasone suppression, and altered responses to corticotropin-releasing hormone [84,85,88,89]. The subsequent literature has supported HPA-axis dysregulation in at least some patients but has also revealed substantial inconsistency across studies, with reports of both increased and reduced basal activity, flattened circadian variation, and attenuated or exaggerated responses to experimental challenge [4,6,20,84].

This heterogeneity makes simple concepts of either “HPA hyperactivity” or “HPA exhaustion” difficult to sustain [4,6,20]. Differences in disease duration, sleep disturbance, psychological stress, physical activity, medication, metabolic factors, sampling time, and the nature of the experimental stressor may all contribute to variation in endocrine findings [4,6,20,84]. Moreover, cross-sectional abnormalities cannot readily distinguish predisposing changes from adaptations to persistent pain, poor sleep, or chronic stress exposure [6,20]. HPA-axis dysfunction is therefore more appropriately viewed as a variable component of altered stress regulation than as a unitary endocrine lesion causing fibromyalgia.

Stress exposure may nevertheless influence pain through interactions among endocrine, autonomic, immune, and central nociceptive systems [6,20,84,85]. Experimental and observational studies indicate that psychosocial stress and negative affect can modify pain responses in fibromyalgia [84,85], while persistent pain itself constitutes a physiological stressor capable of altering stress-system function. The relationship is thus likely to be bidirectional: stress-regulatory abnormalities may enhance vulnerability to symptom amplification, whereas chronic pain and sleep disruption may further reshape HPA-axis responses [4,6,20].

### 9.2. Autonomic Dysregulation

Autonomic symptoms are common in fibromyalgia and may include orthostatic intolerance, palpitations, altered vasomotor regulation, gastrointestinal complaints, and exercise intolerance [85,86,87,88,89,90,91]. Physiological studies have also identified abnormalities in heart-rate variability (HRV), baroreflex regulation, cardiovascular sympathetic activity, and autonomic responses to postural or cognitive challenge [86,87,90,91,92]. The accumulated evidence therefore supports autonomic dysregulation as a relevant component of the syndrome, although neither its direction nor its magnitude is uniform across patients.

Reduced HRV has been one of the most frequently reported findings and is generally interpreted as reduced autonomic flexibility or diminished parasympathetic modulation [20,85,86,87]. Earlier studies also described increased nocturnal sympathetic predominance and abnormal sympathetic responses to orthostatic challenge [86,87]. Direct physiological measurements provide additional evidence: Zamunér et al. demonstrated relationships between resting sympathetic cardiovascular activity and pain intensity in patients with fibromyalgia, including associations involving muscle sympathetic nerve activity and baroreflex indices [92]. These observations support a relationship between autonomic regulation and pain but do not establish persistent generalized sympathetic hyperactivity as the defining autonomic abnormality of fibromyalgia.

Indeed, more recent evidence indicates a more complex pattern. Autonomic abnormalities may involve increased sympathetic predominance under some resting conditions together with impaired or insufficient dynamic autonomic recruitment during challenge [91]. This distinction is important because a system can display altered basal sympathovagal balance while simultaneously responding inadequately to orthostatic or other physiological demands. Autonomic dysfunction in fibromyalgia is therefore better conceptualized as impaired regulatory flexibility than simply as “sympathetic overactivity” [91,92].

Peripheral autonomic small fibers provide an additional level of complexity. As discussed in Section 7, Falco et al. demonstrated reduced innervation of sweat glands and piloerector muscles in patients with fibromyalgia-associated small-fiber pathology [74]. Importantly, however, the degree of autonomic small-fiber loss did not correlate with the severity of autonomic symptoms [74]. Structural peripheral autonomic abnormalities therefore occur in a subset of patients but cannot by themselves account for the broader dysautonomic phenotype.

This central–peripheral distinction has been clarified further by recent cardiovascular autonomic testing. Falco et al. studied patients with fibromyalgia and orthostatic intolerance using head-up tilt, Valsalva maneuver, deep breathing, HRV analysis, and skin biopsy [91]. Compared with healthy controls, patients showed reduced HRV, diminished parasympathetic modulation, altered sympathetic predominance, and impaired dynamic sympathetic recruitment during orthostatic challenge [91]. Patients with concomitant SFP showed greater abnormalities across several autonomic measures and more pronounced tachycardic responses and orthostatic symptoms, yet autonomic dysfunction was not restricted to the SFP subgroup [91]. The findings therefore support a model in which central autonomic network dysfunction is partly modulated by peripheral small-fiber damage rather than fully explained by it [91].

A related line of evidence links autonomic symptom burden to central sensitization. In a large cohort of patients evaluated for chronic orthostatic intolerance, Novak et al. found that individuals meeting questionnaire criteria for central sensitization reported substantially greater autonomic symptom severity, together with greater orthostatic reductions in cerebral blood-flow velocity and more orthostatic tachycardia, even though the frequency of frank autonomic failure was similar in those with and without central sensitization [93]. These findings do not establish a fibromyalgia-specific circuit mechanism, but they support the interpretation that heightened central sensory gain can amplify the clinical expression of dysautonomia independently of the degree of objective autonomic failure or peripheral autonomic fiber loss [74,91,93].

This interpretation provides a useful mechanistic bridge between peripheral and central findings. Autonomic SFP may contribute to impaired postganglionic sympathetic function in some patients [74,91], while abnormalities in central autonomic regulation may affect cardiovascular adaptation, arousal, pain modulation, and stress reactivity across a broader proportion of the fibromyalgia population [85,86,87,88,89,90,91]. The relative contribution of these mechanisms is likely to vary across individuals, reinforcing the need to distinguish autonomic symptoms, objective autonomic dysfunction, and structural autonomic small-fiber abnormalities rather than treating them as interchangeable phenomena.

### 9.3. Sleep Dysregulation

Sleep disturbance is a core component of fibromyalgia and interacts bidirectionally with pain, fatigue, stress physiology, and autonomic regulation [1,4,6,84,85]. Nonrestorative sleep, sleep fragmentation, and altered sleep architecture have long been described in the syndrome, while autonomic studies during sleep have also demonstrated reduced parasympathetic modulation in at least some patients [85,86,87]. Sleep disturbance should therefore not be regarded simply as a secondary consequence of pain.

The relationship between sleep and nociception is likely to operate in both directions. Persistent pain can disrupt sleep continuity and restorative sleep, whereas inadequate or fragmented sleep can increase pain sensitivity, fatigue, affective distress, and vulnerability to stress [4,6,20,84]. Sleep disruption may additionally interact with HPA-axis rhythmicity and autonomic regulation, creating a reinforcing loop in which pain, arousal, and impaired physiological recovery sustain one another [4,20,84,85]. However, the relative importance of these interactions varies among patients, and sleep abnormalities should be regarded as modulatory mechanisms rather than evidence of a single sleep-driven pathogenesis.

This bidirectional model is particularly relevant to mechanistic heterogeneity. In some patients, severe sleep disturbance may act as an important amplifier of nociceptive gain and daytime symptom burden, whereas in others it may be predominantly secondary to pain, mood disturbance, autonomic dysregulation, or comorbid sleep disorders [1,4,6,20]. Sleep phenotyping may therefore provide clinically relevant information but does not define a distinct fibromyalgia mechanism in isolation.

### 9.4. Gender Differences, Sex Hormones and Reproductive Transitions

Fibromyalgia exhibits a clear female predominance, although the magnitude of the sex difference varies substantially according to the diagnostic criteria applied [5,7,8,9,94]. Ratios historically reported as 7:1 to 9:1 in clinical cohorts using tender-point criteria are considerably lower (closer to 2–3:1) in population studies using symptom-based criteria [94,95,96]. Jones et al., for instance, documented a shift from 13.7:1 [7] to 2.3:1 [94]. While these findings caution against attributing the sex difference solely to hormonal factors, the consistent female predominance has nonetheless focused research on the influence of estrogen, progesterone, and testosterone on pain processing and central sensitization.

Nevertheless, gonadal hormones influence nociceptive processing, stress regulation, immune signaling, and affective function and may therefore contribute to sex-related variation in pain susceptibility [97,98]. Estrogen has complex, context-dependent effects on nociception, with both pronociceptive and antinociceptive actions reported depending on concentration, receptor subtype, neural circuit, and hormonal state [97,98]. Progesterone and neuroactive metabolites such as allopregnanolone can modulate GABAergic signaling, while androgens have also been implicated in pain regulation [97,98]. These mechanisms establish biological plausibility but do not demonstrate a specific hormonal abnormality as a universal mechanism of fibromyalgia.

Reproductive transitions may be clinically relevant in some women. Fibromyalgia symptoms have been reported to emerge or worsen around menopause, and earlier menopause has been associated with greater experimental pain and non-pain sensitivity in women with fibromyalgia [99]. However, observational associations between menopause and symptom severity cannot establish that estrogen withdrawal initiates fibromyalgia, because age, sleep disturbance, mood, body composition, comorbidities, and other physiological changes accompany the menopausal transition [97,99]. Hormonal influences should therefore be regarded as potential modifiers of pain sensitivity and symptom expression rather than as primary causal explanations.

Overall, stress-system, autonomic, sleep, and hormonal abnormalities are best understood as interacting regulatory influences whose contribution varies across the fibromyalgia population [1,4,6,20]. HPA-axis findings are heterogeneous rather than consistently hyper- or hypoactive; autonomic dysfunction reflects altered cardiovascular and autonomic flexibility rather than simple sympathetic overactivity; peripheral autonomic small-fiber loss contributes in some patients but does not fully account for dysautonomic symptoms; and sleep and hormonal state may further modulate nociceptive gain [74,91,92,94]. Together, these systems can influence pain, fatigue, arousal, and symptom persistence without constituting a single causal pathway. Their heterogeneity makes them particularly relevant to future mechanism-based phenotyping and provides a natural transition to the psychological and cognitive modulators considered in the following section.

## 10. Psychological and Cognitive Modulators of Pain

Psychological and cognitive processes are important modulators of pain, disability, and symptom burden in fibromyalgia [6,57,100,101]. Their relevance should not be interpreted as evidence that fibromyalgia is primarily psychological. Rather, attention, appraisal, emotion, expectation, and behavior interact with nociceptive processing and stress-regulatory systems and can modify how pain is perceived, maintained, and translated into functional impairment [95,100,101]. Within this framework, factors such as pain catastrophizing, hypervigilance, anxiety, depression, psychological inflexibility, and maladaptive coping are best understood as modulators of the fibromyalgia phenotype rather than as unitary causes of the disorder [6,95,96,100,101].

Pain catastrophizing has received particular attention. It encompasses magnification of pain-related threat, persistent rumination, and feelings of helplessness [96,102]. In fibromyalgia, higher catastrophizing has been associated with greater pain, psychological distress, and functional impairment [6,96,100,103]. Network analyses further suggest that catastrophizing is embedded within a broader set of interactions involving hypervigilance, affective burden, sensory sensitivity, health-related quality of life, and physical function rather than operating as an isolated psychological trait [103]. These associations support a role for cognitive appraisal in shaping symptom severity, although their predominantly cross-sectional nature does not establish that catastrophizing initiates nociceptive abnormalities [103].

Attention represents another important modulatory domain. Pain has a strong capacity to capture attentional resources, and chronic hypervigilance can increase the salience of bodily sensations and pain-related information [101,104,105]. In fibromyalgia, hypervigilance has been associated with catastrophizing and poorer health-related quality of life and forms part of a wider psychobiological network linking sensory, cognitive-emotional, and functional dimensions [103]. Such findings are consistent with broader chronic-pain models in which sustained attention to threat can amplify the subjective burden of pain and reinforce distress and avoidance [101,104,105]. However, attentional amplification should not be assumed to account for the underlying sensory hypersensitivity itself.

Fear and avoidance provide an additional pathway through which pain can acquire increasing behavioral impact. The fear-avoidance model proposes that threatening interpretations of pain may promote avoidance of activity, reduced exposure to normal movement, and progressive functional limitation [95]. Although developed primarily in chronic musculoskeletal pain rather than specifically in fibromyalgia, this framework is relevant to patients in whom fear of symptom exacerbation contributes to reduced activity and disability [6,95,100]. These behavioral consequences may subsequently interact with deconditioning, sleep disturbance, mood, and pain-related expectations, creating self-reinforcing cycles without implying that avoidance is the primary origin of pain.

An important question is whether psychological distress explains the somatosensory abnormalities characteristic of fibromyalgia. Available evidence argues against such a reductionist interpretation. In quantitative sensory testing, Rehm et al. found that depression, anxiety, sleep disturbance, and other psychological variables exerted only limited effects on somatosensory findings, whereas signs compatible with central sensitization were more closely related to pain intensity [16]. Thus, psychological comorbidity may modify symptom expression without accounting for the core pattern of altered sensory processing [6,16]. This distinction is essential: biological pain amplification and cognitive-emotional modulation can coexist and interact without one being reducible to the other.

Cognitive dysfunction, commonly described by patients as “fibrofog,” adds another dimension to this interaction [2,4,106]. Difficulties involving working memory, attention, executive control, and processing efficiency have been reported in fibromyalgia [2,106]. Pain itself places demands on attentional resources, and fatigue, poor sleep, affective distress, medication, and persistent cognitive preoccupation with symptoms may further impair performance [2,4,101,106]. Cognitive dysfunction should therefore not be considered a purely psychological symptom or attributed to a single mechanism; it is more plausibly understood as a multidetermined feature emerging from interactions among pain, attention, sleep, fatigue, and emotional state [2,4,106].

Intervention studies provide additional evidence that cognitive-emotional processes are modifiable and clinically relevant. Taub et al. reported that an adapted mindfulness-based stress reduction intervention improved fibromyalgia symptoms, perceived stress, and depression, with mediation analyses implicating changes in psychological inflexibility and pain catastrophizing [107]. These findings support these cognitive processes as potential mechanisms of change, while not proving that they are primary pathogenic mechanisms of fibromyalgia [107]. More broadly, meta-analytic evidence indicates that cognitive-behavioral approaches can improve selected fibromyalgia outcomes [108], consistent with the capacity of psychological interventions to modify coping, appraisal, behavior, and the affective consequences of persistent pain.

Taken together, psychological and cognitive factors are best understood as interacting components of the fibromyalgia pain-regulatory system [6,100,101,103]. They can influence attention to pain, threat appraisal, emotional salience, avoidance behavior, coping, cognitive load, and functional adaptation [95,96,100,101,102,103,104,105], while available evidence does not support the view that they fully explain the syndrome’s somatosensory abnormalities [6,16]. Their contribution is therefore modulatory and amplificatory rather than exclusively causal, and its magnitude is likely to vary substantially between individuals.

This interpretation is particularly important for mechanism-based phenotyping. Some patients may show prominent catastrophizing, affective distress, hypervigilance, avoidance, or cognitive burden, whereas these dimensions may be less influential in others [16,103]. Psychological and cognitive measures may therefore contribute to characterization of clinically meaningful profiles, but they should be integrated with sensory, neurobiological, autonomic, peripheral, and neuroimmune findings rather than used to define an isolated “psychological subtype.” This integrated perspective provides a natural transition to the broader question of mechanistic heterogeneity considered in the following section.

## 11. Mechanistic Heterogeneity and Candidate Phenotypes

Fibromyalgia is clinically and biologically heterogeneous rather than pathophysiologically uniform [1,16,17,18,19,20,109,110]. Patients differ in pain sensitivity, temporal summation, conditioned pain modulation, small-fiber findings, autonomic function, sleep disturbance, psychological burden, and other symptom dimensions [11,15,16,17,18,19,21,103,109]. Quantitative sensory testing illustrates this variability particularly well: distinct somatosensory patterns occur across patients, and mechanical or thermal hypersensitivity may coexist with sensory loss [16]. Small-fiber pathology is likewise present only in a subset of patients and differs quantitatively and phenotypically from classical small-fiber neuropathy [17,19,63,70,72,73], while conditioned pain modulation ranges from effective inhibition to absent inhibition and, in some patients, pain facilitation [11,15,32]. Clinical clustering studies similarly identify groups differing in symptom burden, pain self-efficacy, catastrophizing, emotional distress, and functional impairment [103,109]. Together, these findings argue against a single physiological or clinical abnormality shared uniformly by all patients.

Current evidence instead supports several recurring candidate mechanistic configurations: a profile dominated by central pain amplification; a profile with prominent descending modulatory dysfunction; a profile with greater peripheral nociceptive or small-fiber involvement; a neuroimmune/DRG-associated profile; and a profile in which stress-autonomic, sleep, and cognitive-emotional factors carry greater relative weight [1,15,16,17,18,19,20,63,70,103]. These profiles are not mutually exclusive diagnostic subtypes. Individual patients may express features of several profiles simultaneously, and their relative weighting may change over time. Central amplification may therefore predominate in some patients, whereas altered descending modulation, peripheral afferent input, small-fiber abnormalities, neuroimmune mechanisms, or stress-autonomic and psychobiological factors may contribute more prominently in others [11,15,16,17,18,19,20,32,63,70,76,77,103].

Importantly, the evidential strength supporting these profiles is unequal. Central amplification, altered descending pain modulation, small-fiber abnormalities, and clinical psychological clustering are supported by direct human data [11,15,16,17,19,32,63,70,103,109]. By contrast, more specific constructs such as a DRG-directed immune phenotype remain emerging and are supported by a combination of human association studies and experimental passive-transfer evidence [76,77]. The distinction between an observed phenotype, a mechanistic hypothesis, and a prospectively validated subtype should therefore be preserved.

No single biomarker currently identifies fibromyalgia or partitions patients into validated mechanistic classes [1,17,18,19]. Measures such as QST, CPM, skin biopsy, corneal confocal microscopy, autonomic testing, neuroimaging, immune markers, and psychological profiling may instead provide complementary information about different components of the phenotype [1,16,17,19,46,63,66,70,103]. Mechanism-based stratification should therefore presently be viewed as a research framework rather than an established clinical classification. Future studies will need to determine whether reproducible multidimensional profiles show sufficient stability, prognostic value, or predictive value for treatment response. The proposed candidate profiles and their current evidential support are summarized in Table 1.

## 12. Translational Implications

The mechanistic heterogeneity of fibromyalgia has important translational implications, but current evidence does not support routine treatment selection on the basis of validated biological subtypes. Rather than assuming that individual mechanisms map directly onto specific therapies, sensory, neurobiological, peripheral, autonomic, sleep, and cognitive-emotional features may eventually contribute to multidimensional patient stratification [1,13,16,17,18,19,20]. The candidate profiles summarized in Table 1 should therefore be regarded as hypothesis-generating frameworks rather than as treatment algorithms.

Current management already reflects the multilevel nature of the syndrome. Pharmacological approaches that modify monoaminergic transmission or neuronal excitability, together with exercise, education, and psychological interventions, can influence partially different dimensions of pain regulation, physical function, sleep, coping, and disability [6,13,14,108,111,112,113,114]. However, therapeutic efficacy should not be interpreted as proof of a corresponding primary pathogenic defect. Response to a serotonin–noradrenaline reuptake inhibitor does not establish a causal monoamine deficiency, just as response to a centrally acting anticonvulsant does not demonstrate that altered excitatory transmission initiates fibromyalgia [6,14,47,48,49,50,51,53,54,111]. Treatment response therefore indicates biological modifiability rather than etiological specificity.

**Figure 1:**
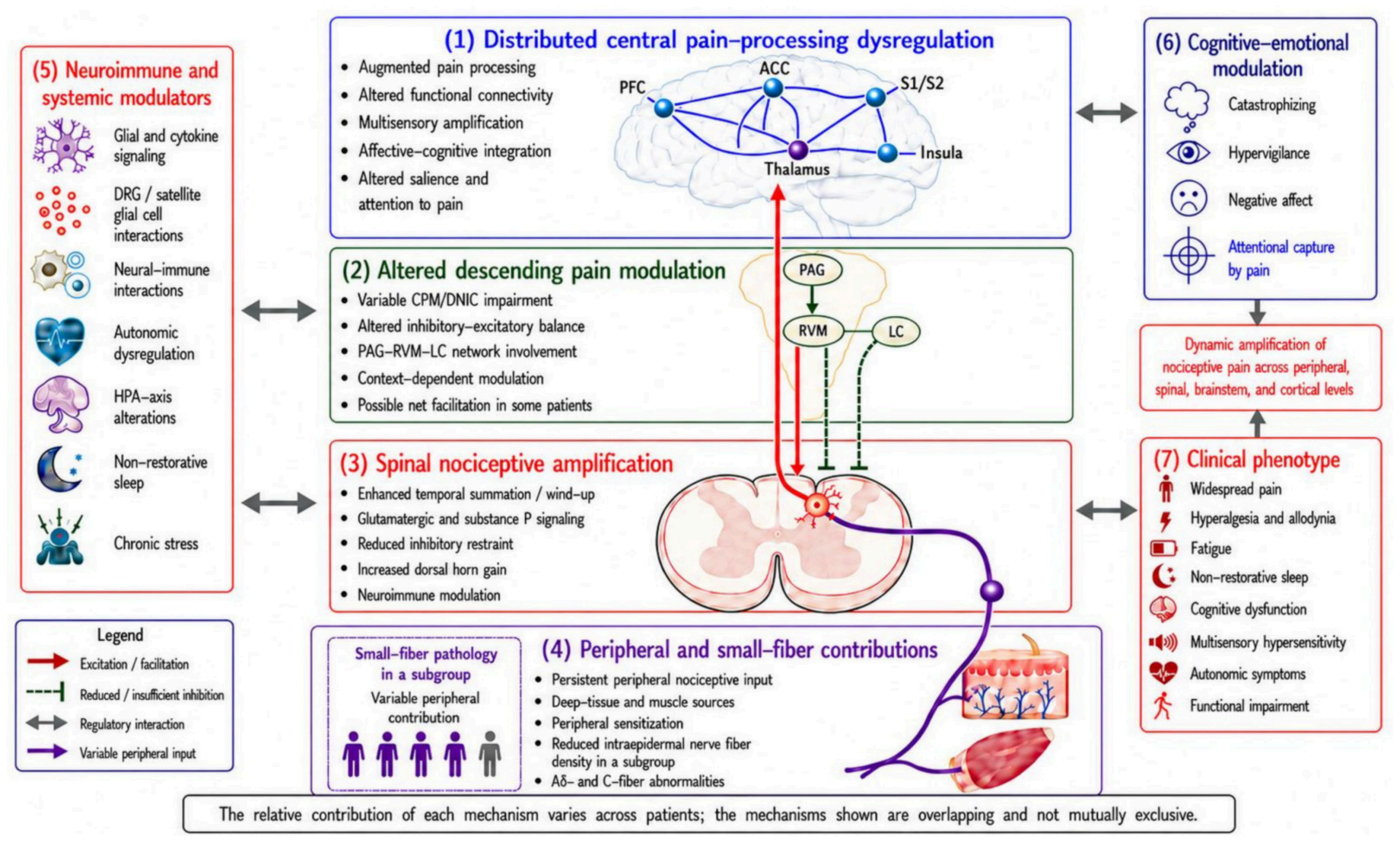
Integrated multilevel model of pain mechanisms in fibromyalgia. Fibromyalgia is represented as a clinically coherent but mechanistically heterogeneous condition arising from interactions among central, brainstem, spinal, peripheral, neuroimmune, autonomic, sleep-related, and cognitive-emotional processes. Distributed central pain-processing dysregulation is illustrated by altered activity and connectivity within pain-related cortical and subcortical networks. Descending modulOKation is represented through the PAG–RVM–LC system, with continuous red pathways indicating facilitatory/excitatory influences and green dashed pathways terminating in inhibitory bars indicating reduced or insufficient descending inhibition. At the spinal level, increased nociceptive gain, enhanced temporal summation, excitatory neurotransmission, and reduced inhibitory restraint contribute to amplification of incoming nociceptive signals. Peripheral input is shown in purple, illustrating variable afferent drive from skin and muscle, together with small-fiber pathology in a subset of patients. Neuroimmune and systemic modulators, including glial and cytokine signaling, DRG–satellite glial cell interactions, autonomic dysregulation, HPA-axis alterations, sleep disturbance, and chronic stress, are positioned as cross-level regulatory influences. Cognitive-emotional modulation interacts primarily with distributed central pain-processing networks, while the resulting multilevel amplification contributes to the shared clinical phenotype, including widespread pain, hyperalgesia and allodynia, fatigue, non-restorative sleep, cognitive dysfunction, multisensory hypersensitivity, autonomic symptoms, and functional impairment. Gray bidirectional arrows indicate reciprocal regulatory interactions rather than direct anatomical projections. The relative contribution of each mechanism is expected to vary among individuals and over time, and the mechanisms shown should therefore be interpreted as overlapping components of a dynamic system rather than as mutually exclusive or fixed biological subtypes. Abbreviations: ACC, anterior cingulate cortex; CPM, conditioned pain modulation; DNIC, diffuse noxious inhibitory controls; DRG, dorsal root ganglion; HPA, hypothalamic–pituitary–adrenal; LC, locus coeruleus; PAG, periaqueductal gray; PFC, prefrontal cortex; RVM, rostral ventromedial medulla; S1/S2, primary and secondary somatosensory cortices.

Emerging approaches—including low-dose naltrexone, neuromodulation, cannabinoids, microbiome-targeted interventions, and immune-oriented strategies—remain insufficiently established for strong mechanism-specific claims [14,76,77,114]. Similarly, no currently available measure, including CPM, quantitative sensory testing, neuroimaging, small-fiber assessment, autonomic testing, or immune biomarkers, has sufficient prospective validation to guide mechanism-specific treatment selection [1,16,17,18,33,46,63,70,80]. The immediate translational priority is therefore to test whether combinations of mechanistic features predict prognosis or treatment response in longitudinal, multimodal studies [1,16,17,18,19]. Until such profiles are validated, multimodal and individualized care remains more defensible than biomarker-based prescribing [13,14,111,112]. Figure 1 summarizes this integrative framework and the dynamic interactions among its major components.

## 13. Conclusions

Fibromyalgia is best understood as a heterogeneous nociplastic pain condition in which altered nociceptive processing emerges from interactions among central, peripheral, neuroimmune, autonomic, endocrine, sleep-related, and cognitive-emotional mechanisms [1,6,11,13,14,16,17,18,19,20]. Central amplification is a major and well-supported component of this framework, but neither central sensitization nor any other single mechanism accounts for the full clinical phenotype or the substantial variability observed between patients.

This integrated model reconciles findings that have sometimes been treated as competing explanations. Central amplification does not exclude peripheral nociceptive contributions; small-fiber abnormalities do not imply that fibromyalgia is a peripheral neuropathy; neuroimmune findings do not establish a universal autoimmune mechanism; and psychological or cognitive factors can substantially modify pain and disability without explaining the syndrome’s core somatosensory abnormalities [6,16,17,19,63,70,76,95,96,100,101,102,103,104,105,107]. Distinguishing association, mechanistic plausibility, and demonstrated causality therefore remains essential.

The current evidence supports partially overlapping candidate mechanistic profiles rather than discrete biological subtypes. Future longitudinal and multimodal studies should determine whether combinations of sensory, neurobiological, peripheral, autonomic, immune, sleep, and cognitive-emotional measures define stable profiles that predict clinical course or treatment response. Until such approaches are prospectively validated, fibromyalgia is best regarded as a clinically coherent but mechanistically non-uniform disorder whose complexity is captured by an integrated, multilevel model [12,13,14].

## Figures and Tables

**Table 1:** Candidate mechanistic profiles in fibromyalgia: clinical characteristics, potential markers, and translational implications.

Candidate Profile	Predominant Mechanisms	Clinical/Sensory Features	Potential Markers	Translational Implications	Current Evidence
Central Amplification	Increased central nociceptive gain; activity-dependent amplification; altered excitatory-inhibitory balance	Widespread hyperalgesia; enhanced temporal summation; prolonged after-sensations; multisensory hypersensitivity	QST; temporal summation; pain-related neuroimaging; glutamatergic measures	Supports strategies aimed at reducing central excitability and strengthening multimodal pain regulation	Strong group-level human evidence; substantial inter-individual variability
Descending Dysregulation	Reduced inhibitory control and/or increased net facilitation within descending pain-modulatory networks	Reduced, absent, or facilitatory CPM; impaired endogenous pain regulation	CPM; cortical-PAG functional connectivity; brainstem imaging	May identify patients in whom modulation of descending monoaminergic or network-level control is especially relevant	Moderate-to-strong human evidence; highly paradigm- and context-dependent
Peripheral/ SFP-associated	Persistent peripheral nociceptive input; structural or functional small-fiber abnormalities	Burning or neuropathic-like pain; sensory abnormalities; dysautonomia; ongoing peripheral pain generators	IENFD; corneal confocal microscopy; QST; autonomic testing	Prompts evaluation for coexisting SFN and may identify patients with a greater peripheral contribution to nociceptive drive	Strong evidence for SFP in a subset; causal role in fibromyalgia remains unresolved
Neuroimmune/ DRG-associated	IgG-related sensory-ganglion signaling; SGC-nociceptor interactions; peripheral and central neuroimmune mechanisms	Pain with possible small-fiber and neuroimmune features; phenotype not yet clinically defined	Anti-SGC IgG; DRG-binding IgG; emerging immune signatures	Potential future basis for immune-oriented stratification or targeted therapies in selected patients	Emerging human evidence combined with experimental passive-transfer evidence; not yet a validated subtype
Stress-autonomic/psychobiological	Altered stress and autonomic regulation; sleep disruption; cognitive-emotional amplification	Dysautonomia; fatigue; nonrestorative sleep; catastrophizing; hypervigilance; affective distress	HRV and autonomic testing; sleep measures; psychological profiling	Supports multidimensional targeting of sleep, stress regulation, behavior, coping, and autonomic function	Substantial human evidence, but heterogeneous and not specific enough to define a discrete subtype

Note: The proposed profiles are not mutually exclusive and have not been prospectively validated as biological subtypes. Individual patients may express features of several profiles, and their relative weighting may vary over time. Abbreviations: CPM, conditioned pain modulation; DRG, dorsal root ganglion; HRV, heart rate variability; IENFD, intraepidermal nerve fiber density; PAG, periaqueductal gray; QST, quantitative sensory testing; SFP, small-fiber pathology; SGC, satellite glial cell; SFN, small-fiber neuropathy.

## Data Availability

No new data were created or analyzed in this study. Data sharing is not applicable to this article.

## Abbreviations

The following abbreviations are used in this manuscript:

ACR	American College of Rheumatology
ASIC	Acid-sensing ion channels
ATP	Adenosine triphosphate
BOLD	Blood-oxygen-level-dependent
CC BY	Creative Commons Attribution
CPM	Conditioned pain modulation
DNIC	Diffuse noxious inhibitory controls
DRG	Dorsal root ganglia
DRt/SRD	Dorsal reticular nucleus/Subnucleus reticularis dorsalis
fMRI	Functional magnetic resonance imaging
GABA	γ-Aminobutyric acid
HPA	Hypothalamic–pituitary–adrenal
HRV	Heart-rate variability
ICVS	Life and Health Sciences Research Institute
IENFD	Intraepidermal nerve fiber density
IgG	Immunoglobulin G
IL-6	Interleukin-6
IL-8	Interleukin-8
LC	Locus coeruleus
LTP	Long-term potentiation
MBSR	Mindfulness-based stress reduction
mGluR(s)	Metabotropic glutamate receptor(s)
NK1	Neurokinin-1
NMDA	N-methyl-D-aspartate
P2X	Purinergic P2X receptors
PAG	Periaqueductal gray
PET	Positron emission tomography
PRISMA	Preferred Reporting Items for Systematic Reviews and Meta-Analyses
QST	Quantitative sensory testing
RVM	Rostral ventromedial medulla
SFP	Small-fiber pathology
SFN	Small-fiber neuropathy
SGC	Satellite glial cells
TNF	Tumor necrosis factor
TRPV1	Transient receptor potential vanilloid 1
TSPO	18-kDa translocator protein
